# Recent Progress in Technologies for Tactile Sensors

**DOI:** 10.3390/s18040948

**Published:** 2018-03-22

**Authors:** Cheng Chi, Xuguang Sun, Ning Xue, Tong Li, Chang Liu

**Affiliations:** 1 State Key Laboratory of Transducer Technology, Institute of Electronics Chinese Academy of Sciences, Beijing 100190, China; chicheng15@mails.ucas.ac.cn (C.C.); sunxuguang16@mails.ucas.ac.cn (X.S.); tli@mail.ie.ac.cn (T.L.); 2 School of Electronic, Electrical, and Communication Engineering, University of Chinese Academy of Sciences, Beijing 100190, China

**Keywords:** tactile sensor, technologies progress review, humanoid robot, MIS

## Abstract

Over the last two decades, considerable scientific and technological efforts have been devoted to developing tactile sensing based on a variety of transducing mechanisms, with prospective applications in many fields such as human–machine interaction, intelligent robot tactile control and feedback, and tactile sensorized minimally invasive surgery. This paper starts with an introduction of human tactile systems, followed by a presentation of the basic demands of tactile sensors. State-of-the-art tactile sensors are reviewed in terms of their diverse sensing mechanisms, design consideration, and material selection. Subsequently, typical performances of the sensors, along with their advantages and disadvantages, are compared and analyzed. Two major potential applications of tactile sensing systems are discussed in detail. Lastly, we propose prospective research directions and market trends of tactile sensing systems.

## 1. Introduction

Over the past two decades, robots have gathered much attention and have been playing a more important role in the industrial scene and in routine life. Taking the human being as a paradigm, mature technologies and significant achievements in imitating visual and auditory functions have been demonstrated [1,2]. Robots integrated with tactile sensors could help obtain tactile information, such as the magnitude and direction of a contact force, temperature, humidity, and texture, which is essentially significant for stable grasps, path planning, and obstacle avoidance in unstructured environments [3]. Furthermore, object manipulation tasks and safe human–machine interaction both require reliable tactile sensors [4]. It is, thus, necessary for robots to obtain tactile sensing capabilities to manipulate objects precisely and properly in an unstructured and complex environment.

**Figure 1:**
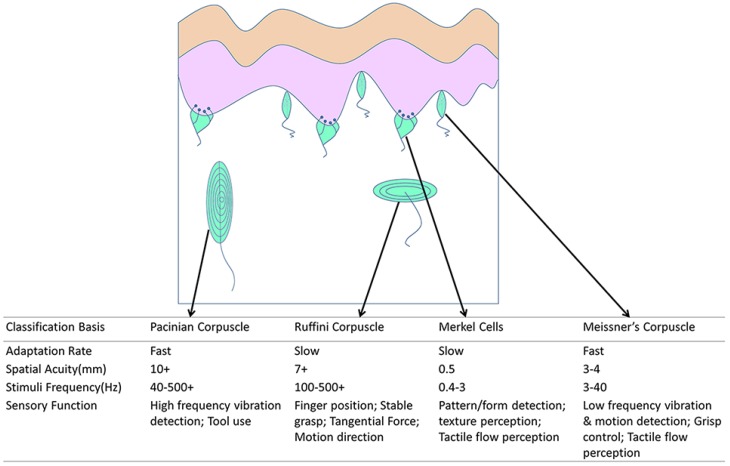
Illustration of the distribution and classification of various mechanoreceptors [10,13,14,15,16,17,18].

The human tactile system operates through quantities of receptors to obtain contacted tactile information, and main receptors include mechanoreceptors, thermoreceptors, and nocioceptors [5,6]. Mechanoreceptors detect pressure and vibration and can be divided into four kinds: Meissner corpuscles, Merkel cells, Ruffini endings, and Pacinian corpuscles [7,8]. The physical position and classification of mechanoreceptors are shown in Figure 1. The spatial resolution varies across the body, is highest at the fingertips (1 mm [9]), and is lowest at the belly (30 mm [10]). Additionally, the temporal resolution reaches up to 700 Hz [11]. Properties of the human tactile system create basic design demands for tactile sensors. For instance, tactile sensors should measure a three-dimensional force between 0.01 and 10 N with a response time of less than 1 ms [12]. For body sites such as the fingertips, the spatial resolution should be about 1 mm; for less sensitive sites such as the palm and shoulders, it could be as high as 5 mm [12]. However, spatial resolution of tactile sensors should achieve 1 mm.

Tactile sensors have been developed and actualized for almost 40 years. As early as the 1970s, Kinoshita et al. used piezoelectric sensing arrays to form a visual–tactile symbiotic system and assembled the array onto a robot hand [19]. In the 1980s, Raibert et al. developed a tactile sensor array using conductive rubber and metal electrodes on the surface of an integrated circuit [20]. Up to the 1990s, instead of rigid material, flexible and stretchable materials became a new area of interest [21,22,23,24]. Ohtsuka et al. integrated a piezoelectric tactile sensor with a thoracoscopic detector for the localization of small invisible nodules in the lung, which is a representative application in minimally invasive surgery (MIS) [25]. Especially in the 21st century, the pursuit to mimic the complex function of human skin has attracted much interest, limited to the sensing of not only pressure but also temperature, humidity, hardness, viscosity, and self-healing [26,27,28,29,30,31,32,33,34,35,36,37,38,39,40,41,42,43,44,45,46,47]. Except for those basic parameters, comprehensive information from contact objects, such as texture [48,49], shape [50,51,52], and slip [48,53,54,55], is also required. Engel et al. realized the integration of a polymer micromachined tactile array using polymer materials and metal thin films to detect the hardness, thermal conductivity, temperature, and surface contours of a contact object [56]. Although this research has lasted for 40 years, experimental results have still struggled to gain prominence to satisfy customers’ requirements. This could be attributed to the lack of dexterity, flexibility, and robustness [57].

This paper provides a review of state-of-the-art technologies in tactile sensor research. Various transduction mechanisms and their advantages and disadvantages are highlighted in Section 2; then, we introduce several representative applications in Section 3; additionally, an outlook of future development is described in Section 4; finally, conclusions are offered in Section 5.

## 2. Transduction Mechanisms

Tactile sensors have been researched using nearly all known modes of transduction methods, including capacitive, piezoresistive, piezoelectric, optical, and magnetic. Detailed classification and analysis of state-of-the-art structures are presented in this section. A comparison of various transduction principles in the past five years of publication is shown in Table 1. Additionally, we provide a summary of advantages and disadvantages of the principles in Table 2.

### 2.1. Capacitive Tactile Sensors

Capacitive sensing is one of the most common principles used in robotic tactile sensing. The capacitance of parallel plate capacitor is expressed as

(1) $$C=\varepsilon_{0}\varepsilon_{r}\frac{A}{d}$$

where A is the overlapping area of the two electrodes, ε_0_ is the permittivity of vacuum, ε_r_ is the relative permittivity of the dielectric layer, and d is the distance between the electrodes. Most researchers detect the changes in A or d to measure the pressure/force applied on the sensor [81]. Due to the characteristics of high sensitivity and resolution, capacitive sensors are appealing to researchers. Recently, most studies focus on the design of the dielectric layer and electrode structure. We will discuss these two schemes in a detailed manner in Section 2.1.1 and Section 2.1.2.

#### 2.1.1. Design of the Dielectric Layer

In recent research, it has been a common solution to fabricate a compressible dielectric layer to increase the sensitivity of the capacitive tactile sensor [62,82]. Among the materials that have been researched, polydimethylsiloxane (PDMS) is typical [83]. It is well-known that PDMS possesses good elastic properties and biomedical compliance with human tissue [84] and living cells [85]. Thus, PDMS exhibits high performance as a material of dielectric thin films in capacitive tactile sensors [82].

**Figure 2:**
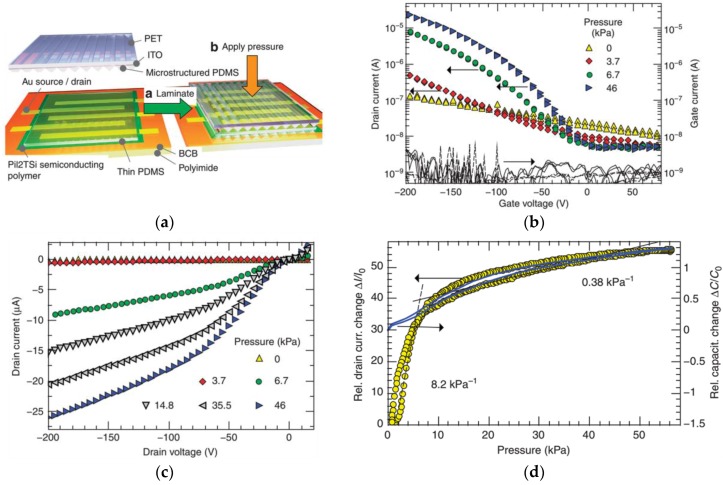
Flexible pressure-sensitive organic thin film transistors (OTFTs). (**a**) Schematic of the fabrication step; (**b**–**d**) electric characteristics of the OTFTs. Reprinted from [86], copyright (2013), with permission from Nature Publishing Group.

Capacitive tactile sensors with non-patterned PDMS dielectric layers generally exhibit large measurement ranges, but low sensitivity. To solve this problem, Schwartz et al. proposed a flexible pressure-sensitive polymer transistor using a microstructural PDMS dielectric layer [86]. An organic thin film transistor (OTFT) was assembled using lamination techniques. First, the bottom source and drain electrodes and the polymer semiconductor were fabricated on a divinyltetramethyldisiloxane bis(benzocyclobutene) (BCB)-coated polyimide film. Polyisoindigobithiophene-siloxane (PiI2T-Si) was adopted to compose the semiconductor film because of its high mobility and resistance to most solvents as an annealed film [87]. To increase the sensitivity, a microstructured V-shape-groove PDMS dielectric layer on an indium tin oxide (ITO)-coated polyethylene terephthalate (PET) film was further proposed [82]. Upper and lower layers were laminated together, with the groove structures aligned parallel to the channel. The schematic of the fabrication step is shown in Figure 2a, and the electric characteristics of the OTFT are shown in Figure 2b–d. In comparison with those capacitive tactile sensors with monolithic PDMS, the reported OTFTs exhibit a significant increase in performance with a maximum sensitivity of 8.4 kPa^−1^, a response time of less than 10 ms, and a power consumption of less than 1 mW. However, the high sensitivity and stability are obtained under the extremely high source drain and source-gate voltage (both are −200 V), which are difficult to achieve in practice.

**Figure 3:**
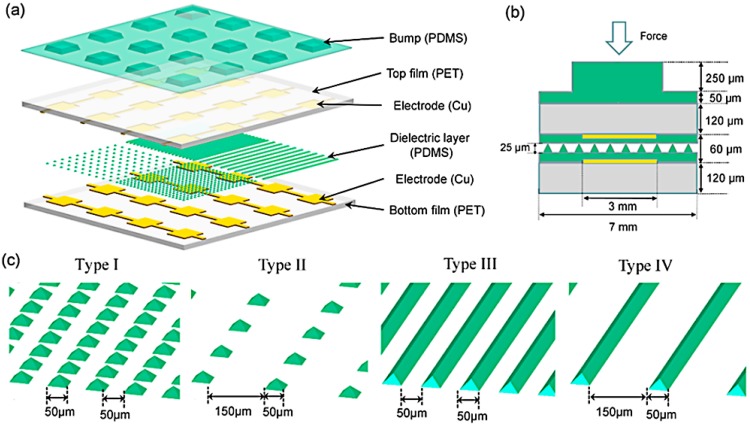
(**a**) Schematic diagram of the capacitive tactile sensing array; (**b**) cross-section view of one sensing unit; (**c**) schematic diagram of different geometries of the microstructures on the polydimethylsiloxane (PDMS) layer. Reprint from [89], copyright (2016), with permission from MDPI AG.

Apart from the V-shaped groove, a pyramid is another popular structure with increasing interest, not only used in capacitance but also in piezoresistivity [88]. A pyramid structure could decrease the visco-elastic creep to the utmost extent, thereby storing and releasing the energy reversibly and thus minimizing the problems associated with the visco-elastic behavior [82]. Ji et al. compared the performance of the two structures when they were integrated into a tactile array [89]. The top and bottom Cu electrodes are patterned on the PET substrates and the 60-µm-thick PDMS film with the microstructure is assembled between the electrodes. Additionally, a PDMS bump contact layer is placed on the top to concentrate the force intensity. The schematic and cross-section view are shown in Figure 3a,b. Experimental results indicated that, within the force range of 0–1 N, the sensitivity of the pyramid-structured units is five times higher than those of the V-shape-structured units, which has also been demonstrated via the finite element modeling method [60]. They also researched the effect of different feature space of the PDMS structure as shown in Figure 3c. Pyramid and V-shape groove structures were both fabricated with a feature space of 50 µm and 150 µm. Tactile units with a feature space of 150 µm exhibit a three times higher sensitivity than those with a feature space of 50 µm.

**Figure 4:**
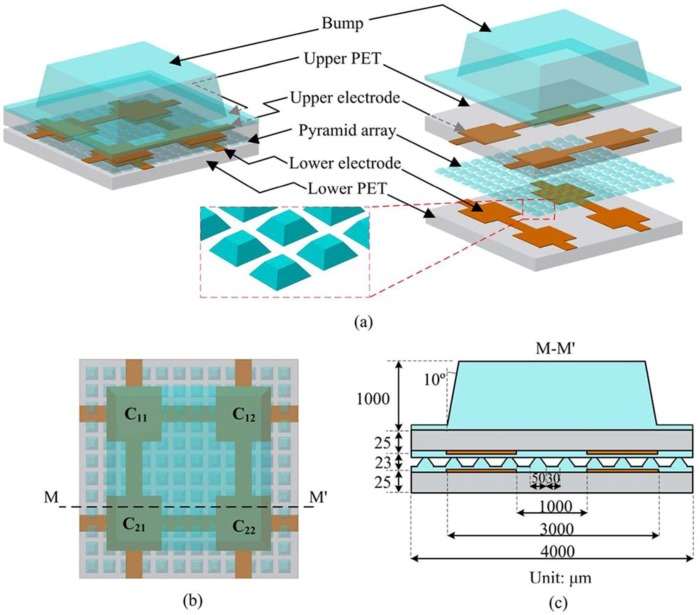
Tactile sensor array with a truncated PDMS pyramid array as a dielectric layer. (**a**) Schematic view of the capacitive tactile sensor unit; (**b**) top view of the capacitive tactile sensor unit; (**c**) cross-section view along M–M’ © [2015] IEEE. Reprinted, with permission, from [62].

Although capacitive tactile sensors with a pyramid PDMS dielectric layer possess high sensitivity, a fast response speed, and a small relaxation time, the tiny contact area between the pyramids and the electrodes will reduce the robustness when shear force occurs. To improve this problem, Liang et al. proposed a three-dimensional flexible capacitive tactile sensor array embedded with a truncated PDMS pyramid array as a dielectric layer [62]. When three-dimensional force is applied onto the tactile sensor, magnitudes of the forces on the *x*-axis, the *y*-axis, and the *z*-axis could be measured according to the difference and co-effect between C11, C12, C21, and C22. The truncated pyramid structure allows the sensor to achieve high sensitivity and good robustness simultaneously. An illustration of the sensor is shown in Figure 4. The reported sensitivity in the normal direction S_z_ = 67.2%/N (the “%/N” and “kPa^−1^” below are common sensitivity units) at a force range of 0–0.5 N, while S_z_ = 7.7%/N at a force range of 0.5–4 N. Typical sensitivity in the shear direction is S_x_ = 58.3%/N and S_y_ = 57.4%/N at a force range of 0–0.5 N.

There are also some other designs that have served as the dielectric layer of a capacitive tactile sensor, such as an air gap [90], nano-needles [91], and fluid [92]. These materials will respond intensely even if the contact force is very small. However, they will take more time to recover to their initial states. The response time will increase significantly, which is harmful to continuous force measurement.

#### 2.1.2. Design of the Electrode

The most common method of measuring three-dimensional force in dielectric layer designs is measuring the difference and co-effect of four sensing cells in one unit, as reported in [62]. Due to the visco-elastic effect and structure of the bump layer, the sensitivity and accuracy of shear force is much lower than that of the normal force. In order to solve this problem, optimizing the electrode design is an effective way. Diverse shapes of electrodes can enlarge the capacitance variation range in response to shear force, leading to better sensitivity and accuracy. Finger-like electrodes are widely used in such sensors.

**Figure 5:**
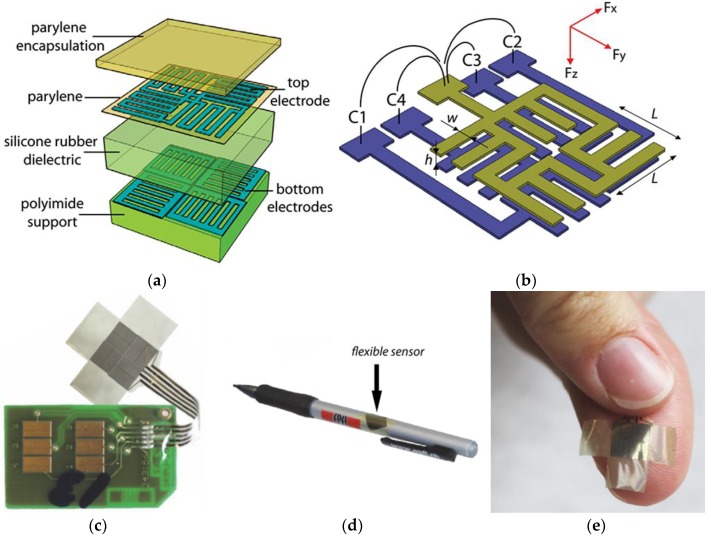
Flexible polymer-based three-axial force sensor. (**a**,**b**) A conceptual view of the sensor; (**c**) sensor and condition circuit; (**d**,**e**) the sensor conforming to a pen and fingertip. Reprinted from [59], copyright (2012), with permission from IOP Publishing.

Dobrzynska et al. developed a flexible polymer-based three-axial capacitive tactile sensor, which integrated three kinds of polymers with standard metallization techniques [59]. A conceptual view of the sensor is shown in Figure 5a,b, and photographs of the sensor are shown in Figure 5c–e. Each sensing unit detecting shear force consists of two series of finger-shaped electrodes perpendicular to each other. When a normal force F_z_ is applied to the sensor, the elastic dielectric is compressed, and all four capacitors increase their capacitance value. When a shear force F_x_ is applied, the elastic dielectric deforms along the *x*-axis, so that capacitors C1 and C3 change their value, nevertheless the remaining two capacitors (C2 and C4) are only sensitive to the *y*-axis shear force and remain unresponsive. The sensitivity of the normal force can reach up to 0.024 kPa^−1^ for pressures less than 10 kPa, and the measured sensitivity for higher pressures is 6.6 × 10^−4^ kPa^−1^. Typical measured shear force sensitivity is 2.8 × 10^−4^ kPa^−1^.

**Figure 6:**
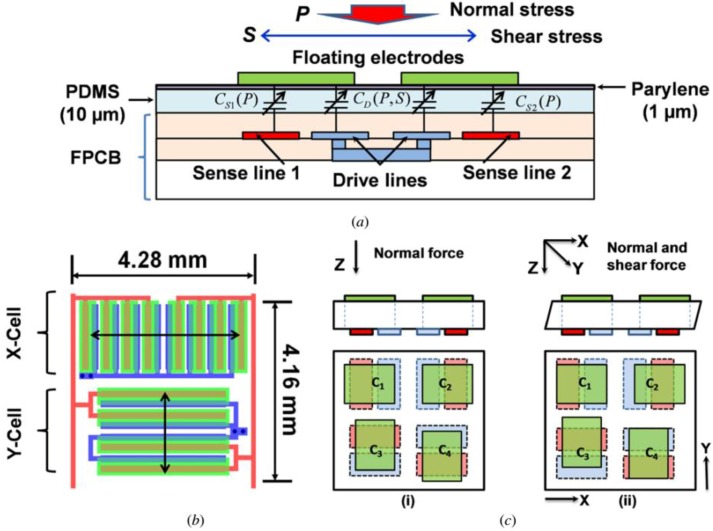
(**a**) Cross-section view of a sensing cell; (**b**) illustration of the electrodes in the top view; (**c**) the sensing principle of three-axis force. © [2012] IEEE. Reprinted, with permission, from [93].

Experiments infer that the upper electrode is prone to emerging metallization breaks after hundreds of cycles. The capacitance value of the sensor will undergo radical change. Surapaneni et al. reported a three-axis capacitive tactile sensor with floating electrodes [93]. Since the floating electrodes do not require any wiring, the sensor is unsusceptible to metallization breaks. In one sensing unit, the capacitance is split into two series: one is between the sense fingers and corresponding floating electrodes and the other is between the floating electrodes and the corresponding drive electrodes, as shown in Figure 6a. When applying a normal force onto the sensor, the capacitance value of the four parts increase equally, as shown in Figure 6c(i). When shear force is applied, the capacitance value of one part increases and that of the other part decreases, as shown in Figure 6c(ii), which can increase sensitivity. The equal sensitivity in the *x*- and *y*-axes is achieved by designing the size and number of finger electrodes.

Although capacitive tactile sensors exhibit high sensitivity and spatial resolution, as reported in [59,62,86,89,93], obvious drawbacks still exist. For instance, the inherent stray capacitance problem makes capacitive tactile sensors restricted in many working scenes.

### 2.2. Piezoresistive Tactile Sensors

The basic principle of this type of sensor is the transduction of force variations into resistance changes. Due to the simplicity of their device design and readout circuit, piezoresistive tactile sensors have recently attracted much interest. As for the choice of the sensing element, the most common approaches are nanocomposites and doped silicon cantilever beams.

#### 2.2.1. Nanocomposites

In recent years, the rapid advancement of nanomaterial and microstructure fabrication has led to the triumphant applications in sensor designs. Tactile sensors using nanocomposites are usually mechanically flexible, robust, and chemically resistant, and these characteristics afford them the potential to be widely used in robotics. According to the principle of sensing, such piezoresistive sensors can be divided into two categories [94]: those that use volume changes in sensitive materials to detect pressure [94,95] and those that take advantages of the change in the contact area at the microscopic scale [96,97,98]. Nanocomposites generally comprise soft polymer matrices and nanoscale conductive fillers embedded in matrices. There are two kinds of conductive fillers widely used in piezoresistive tactile sensors: metal-based and carbon-based fillers.

Typical metallic fillers mainly include Ag [99] and Ni [100]. However, the coherent incompatibility issues between metallic particles and the polymer usually cause failures in the fabrication process, especially in the bonding of thin layers, limiting their application to flexible electronics.

Carbon-based fillers generally consist of carbon black [101], carbon nanotubes (CNTs) [102], and graphene [103]. In comparison with carbon black, CNT-based nanocomposites require lower conductive phase concentration, leading to better mechanical properties of the composite [104]. In addition, CNTs have remarkable electrical and mechanical properties and high aspect ratio, which have rendered CNTs an ideal option for conducting nanocomposites [105,106]. Commercial products, force sensing resistors (FSRs), are also based on this mechanism [107,108]. Various studies have evaluated the sensitivity of polymer/CNT composites for strain sensing [109,110] and pressure sensing [111].

**Figure 7:**
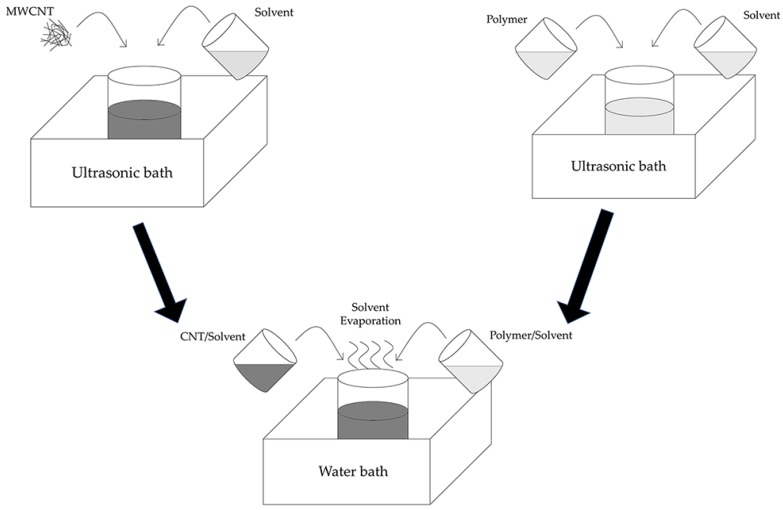
Illustration of ultrasonication process to fabricate polymer/carbon-nanotube (CNT) composites.

A challenging issue with polymer/CNT composites is the proper dispersion of CNTs in the base polymer, which would significantly affect both mechanical and electrical properties. Effective dispersion of CNTs requires that the van der Waals force between nanotubes is overcome [112]. Various approaches have been reported in recent years, such as shear mixing [113], ultrasonication [114], ball milling [115], and micro-bead milling [116]. Ultrasonic processes have attracted significant interest because the procedure is simple and the dispersion effect is extraordinary. In this case, two dispensed solutions containing carbon nanotubes and polymer resin, respectively, share the same organic solvent, and sonication is performed to mix them together. Finally, after the solvent is completely evaporated, CNTs disperse evenly in the polymer. The illustration of this process is shown in Figure 7. Liu et al. reported a detailed comparison of several organic solvents of the MWCNT/PDMS composite, including toluene, tetrahydrofuran (THF), chloroform, and dimethylformamide (DMF) [117]. They compared CNT dispersion, the solubility of the PDMS base polymer, and the effect of PDMS on dispersed CNTs in different organic solvents. According to the experimental results, chloroform performed best among the four tested solution, implying that chloroform can disperse MWCNTs uniformly in PDMS. This assessment method could serve as a paradigm when solvent for other polymers are selected.

**Figure 8:**
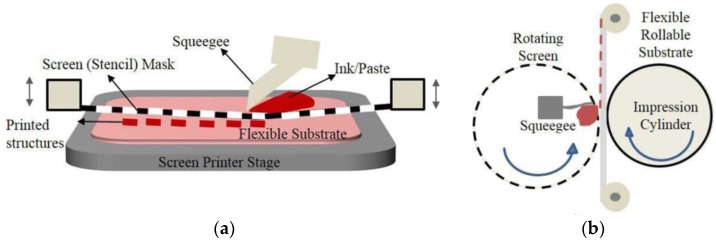
(**a**) Illustration of flatbed screen printing; (**b**) an illustration of rotary screen printing © [2015] IEEE. Reprinted, with permission, from [118].

Khan et al. determined the most common printing technologies for flexible sensors and electronics [118]. Screen printing is the most versatile one and most suitable for printing nanocomposites onto flexible substrates. Several attempts have been made [67,119]. Screen printing has matured and has been applied in industry for quite a long time; for example, metallic interconnects are printed on printed circuit boards (PCBs). Screen printing also exhibits significant reproducibility through different batches [120]. According to the processing methods, screen printing can be divided into flatbed screen printing and rotary screen printing, as shown in Figure 8. A screen, a squeegee, a press bed, and a substrate constitute the main experimental setup. In flatbed screen printing, the squeegee is swept across the screen to transfer the template pattern onto the substrate. Flatbed screen printing has become a powerful tool in laboratories. Furthermore, a rotary screen could replace flatbed screens in the mass production for continuous processing, in which the screen is designed to be cylindrical while the squeegee and ink are placed inside.

#### 2.2.2. Strain Gauge

**Figure 9:**
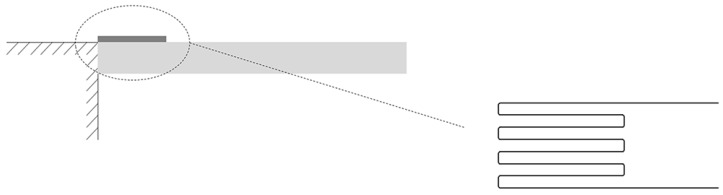
Schematic view of strain gauge.

Strain gauge is a typical way to measure contact force in MEMS piezoresistivitive sensors [121], taking the form of a zig-zag pattern of metallic foil deposited on flexible backing, as described in Figure 9. The electrical resistance will change when the gauge is stretched or compressed. Some of the common choices for gauge material are constantan [122], isoelastic [123], karma [124], and platinum [125], depending on the application. Metallic or alloy gauges generally exhibit high sensitivity and spatial resolution, and benefit from a mature Si-based fabrication process [126]. However, the lack of flexibility is obvious. The strain gauge is most suitably placed on the parallel-jaw gripper of the robot.

**Figure 10:**
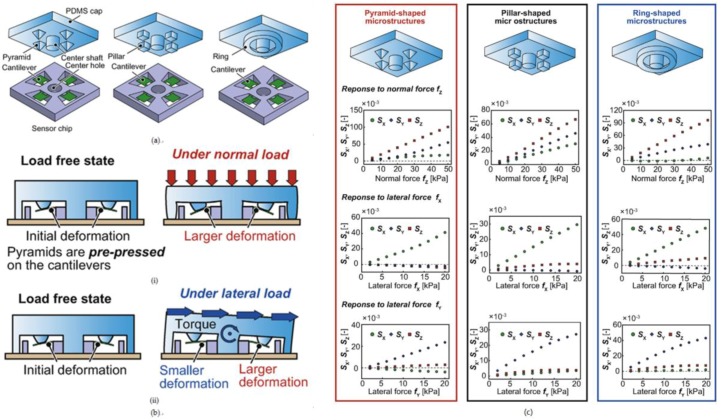
(**a**) Schematic view of the proposed tactile senor with three kinds of convex microstructure; (**b**) schematic view of the sensing mechanism; (**c**) experimental results of the proposed tactile sensor. Reprint from [127], copyright (2014), with permission from Elsevier.

Strain gauges are traditionally used to wrap cantilevers with a flexible polymer, using the polymer as protective and force-conducting media. However, the drawback of this method is that the sensing area and sensitivity may not be enough. To solve this problem, Thanh-Vinh et al. proposed a novel design to increase the sensitivity and sensing area, using an air cavity to displace conventional embedding designs [127]. The air cavity eliminates the limitation of the surrounding elastomer in the conventional design. Moreover, the PDMS cap transformed the entire contact surface into a sensitive site, greatly increasing the sensing area of the original design. The sensing mechanism is shown in Figure 10b. Additionally, they compared three kinds of convex microstructures: a pyramid, a pillar, and a ring, as shown in Figure 10a. Experimental results are shown in Figure 10c. The pyramid-shaped microstructure achieved the highest sensitivity to normal force; however, the ring-shaped microstructure showed the best response to shear force.

#### 2.2.3. Doped Silicon

In comparison with a strain gauge, a doped silicon beam has a higher sensitivity and a wider measurement range. Similar to a strain gauge, a doped silicon beam also suffers from fragile silicon material, so the sensor does not conform to a curved surface.

**Figure 11:**
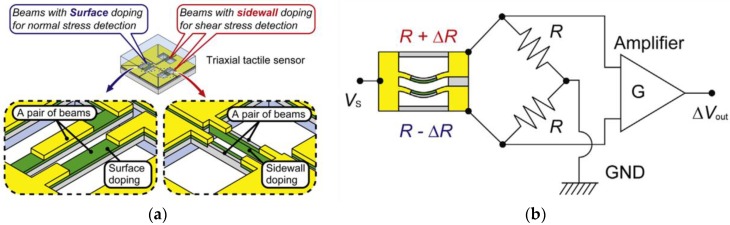
(**a**) Schematic view of the tactile sensor; (**b**) a schematic view of the bridge circuit to measure shear force. Reprint from [128], copyright (2013), with permission from Elsevier.

Okatani et al. developed a triaxial tactile sensor consisting of two pairs of sidewall-doped Si beams for shear force sensing and one pair of surface-doped Si beams for normal force sensing [128], as shown in Figure 11a. While shear force is applied onto the sensor, the pair of sidewall-doped beams that is perpendicular to the shear force direction is bent. As the sensing material on one beam is stretched, the resistance of one beam increases, while the resistance of the other beam decreases. Shear force can be measured via a bridge circuit, as shown in Figure 11b. The later work of their group demonstrated that the sensor could also be used to measure the coefficient of static friction [129].

Piezoresistive tactile sensors exhibit excellent potential in both laboratory results and commercial products. Nevertheless, hysteresis and lack of reproducibility remain the largest impediment to further practical application. According to our experiment results, the resistance value of MWCNT/PDMS prepared in different batches with the same parameters may show a difference of almost 10 times. The diverse resistance values in a tactile sensing array requires a large amount of time to calibrate.

### 2.3. Magnetic Tactile Sensors

Tactile sensors based on magnetism are another kind of tactile sensor that can mimic the mechanosensorial receptors in human fingers. Robustness and a lack of mechanical hysteresis are advantages of such tactile sensors. Magnetic tactile sensors that have been recently developed mainly depend on two operating principles: (1) measurement of the change in magnetic flux or magnetic field intensity by the Hall effect or giant magneto resistance (GMR); (2) use of electromagnetic induction in coupled coils when an external force is applied to them, which results in mechanical deformation. 

#### 2.3.1. Magnetic Field Detection

Generally, tactile sensors based on piezoresistive or capacitive principles have to be made into arrays in order to detect 3D forces. However, magnetic tactile sensors utilizing the Hall effect or GMR to measure the change in magnetic field caused by applied forces can directly detect 3D forces of a single point. This advantage is very important for high-resolution and low-power consumption in tactile sensors fabrication. 

**Figure 12:**
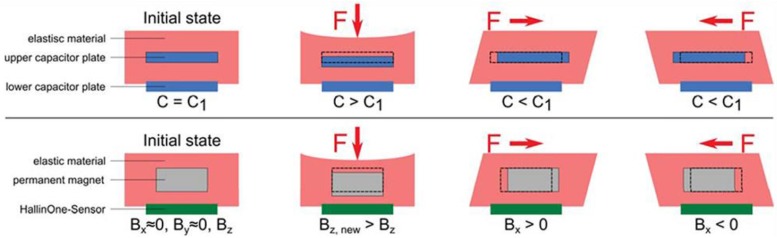
Working principle of a conventional capacitive tactile sensor and the magnetic tactile sensor © [2013] IEEE. Reprinted, with permission, from [130].

**Figure 13:**
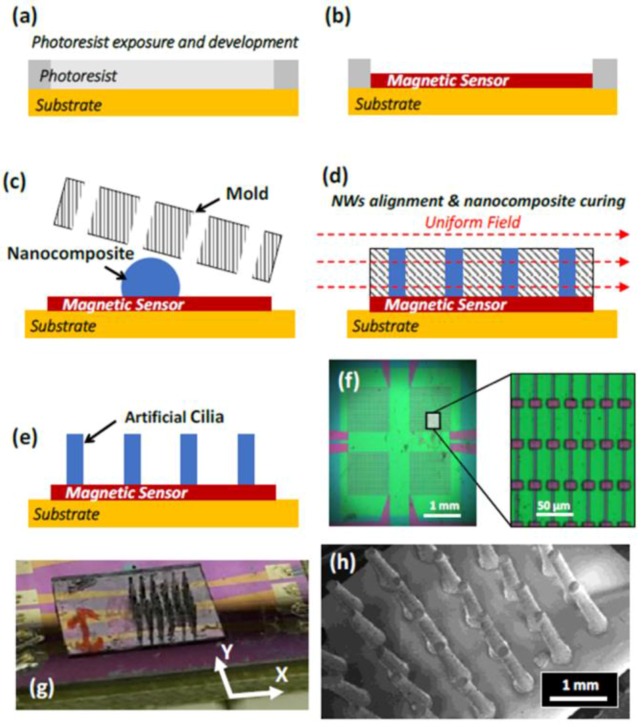
(**a**–**e**) Fabrication process of the tactile sensor. (**f**) Microscope image of the GMR sensor array prior to cilia integration. (**g**) Optical image of the fabricated sensors. (**h**) SEM image of a cilia array. Each cilium is 1 mm in length and 200 μm in diameter © [2016] IEEE. Reprinted, with permission, from [131].

Ledermann et al. proposed a magnetic tactile sensor utilizing a commercial 3D Hall sensor, AS54xx, and embedding a permanent magnet in the elastic material that covers that AS54xx [130]. The working structure of the sensing part that senses the applied forces of their tactile sensors is similar to the structure of conventional capacitive tactile sensors, as shown in Figure 12. The elastic material will deform when external forces are applied, which can change the position of the permanent magnet. The AS54xx can measure the change in the magnetic field vector so that the amplitude and direction of forces can be procured. However, the diameter of the circular PCB in which the 3D Hall sensor AS54xx is fabricated is 9 mm, and the entire sensor prototype is fabricated in a silicon pad with a diameter of 16 mm, which hampers the actualization of a high spacial resolution. Alfadhel et al. recently proposed a kind of tactile sensor using a magnetic nanocomposite hair-like cilia, which can recognize small surface texture changes [41,131]. External 3D forces cause the deflection of cilias resulting in a change in the magnetic stray field of the cilia and the change of the magnetic stray field can be detected by a GMR sensor. The artificial cilia is made of nanocomposites, which are composed of PDMS and iron NWs at a maximum NW/PDMS volume ratio of 14%. Iron NWs, which have high magnetization at remanence, high coercivity, and biocompatibility in the PDMS, are aligned during the fabrication process, which is shown in Figure 13 to serve as nanopermanent magnets. The fabrication process involves a standard lithography process, ion beam deposition, ion milling on a silicon substrate, and PMMA mold techniques. The prototype of this cilia tactile sensor has been used for Braille character reading, and the number of cilia in arrays can be set up flexibly depending on the area or sensitivity required by a given robotic application. Furthermore, the magnetic cilia tactile sensor has extraordinary low power dissipation owing to the use of permanent magnets, which require no power consumption, and the sensor can measure flow 3D forces in liquid environments due to its unique biomimetic structure, which is a great advantage over conventional tactile sensors that only detect solid forces. 

#### 2.3.2. Electromagnetic Induction

Tactile sensors based on the principle of electromagnetic induction essentially utilize Faraday’s law of induction. When the magnetic field in the coil changes, the induction voltage value changes along with the rate of change in magnetic flux [132]. To generate induced voltage in coils due to the changing magnetic field, two approaches are usually employed. One is applying an inconsistent magnetic field, which is mainly produced by the Helmholtz coil to all loop coils. The amplitude of the induced voltage is related to several parameters, such as the excitation frequency, the magnitude of magnetic flux, the number of turns of loops, the area of the coil, and the angle between the plane of coils and the applied magnetic field, which provides broad lines of thinking for researchers in terms of transformation principles. The other approach is setting coils, called excitation coils, coupled with induction coils in each dimension. The coil generating the induced voltage is called detection coil. 

**Figure 14:**
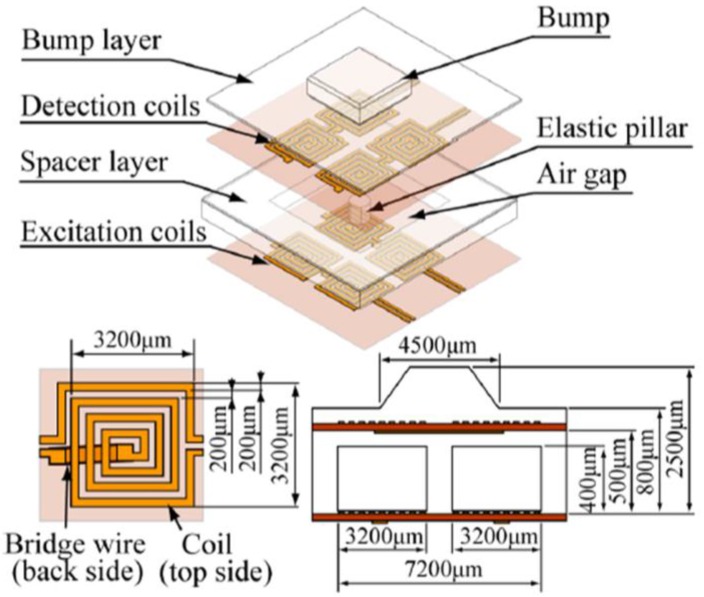
Structure and conformation of the proposed three-dimensional tactile sensor © [2012] IEEE. Reprinted, with permission, from [76].

**Figure 15:**
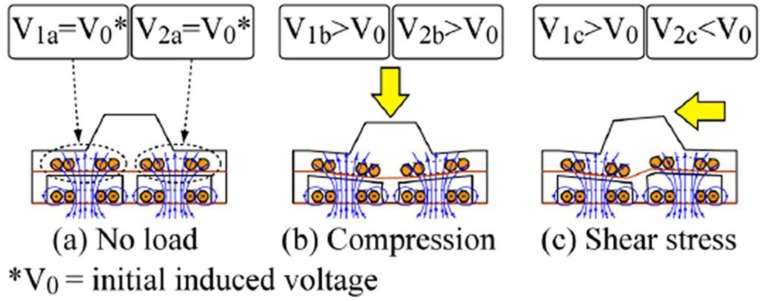
Working principle diagram of the three-dimensional tactile sensor: (**a**) without load; (**b**) under compression; (**c**) under a shear force © [2012] IEEE. Reprinted, with permission, from [76].

Wattanasarn et al. proposed a three-dimensional magnetic tactile sensor using flexible induction coils embedded in elastomeric substrates [76]. The planar coils array in each layer is arranged in two rows and two columns, and a PDMS spacer layer, which has an elastic pillar and air cavity inside, is sandwiched between two layers, as shown in Figure 14. Detection coils generate an induced voltage by detecting the magnetic field produced by the current in the excitation coils. A mechanical deformation occurs when an external force is applied to the bump, as shown in Figure 15, which generates a higher voltages of coils in compressed detection as a result of the truncated distance between detection and excitation coils, and the voltages of the stretched detection coils change inversely for an elongated distance.

Although magnetic induction tactile sensors a good choice for artificial skin in robot application because they are flexible, sensitive, and easy to fabricate, there are still drawbacks that should be taken into consideration. For instance, electromagnetic induction tactile sensors consume more power, in comparison with magnetic tactile sensors that use permanent magnets, because of the difference in current in the excitation coils. Additionally, the eddy current effect and stray capacitance make sensors less reliable and decrease the sensor’s performance in some degree.

### 2.4. Piezoelectric Tactile Sensors

Tactile sensors based on piezoelectric principles transduce external force or pressure to output voltage proportionally. The most essential elements of this kind of tactile sensors are piezoelectric materials, which generate charges under the circumstances of being subjected to external force/pressure. Thus, piezoelectric materials used for dielectric materials with a certain thickness and area can be regarded as capacitances approximately when dynamic forces are applied to them. Polyvinylidene fluoride (PVDF) and its copolymers are most widely used as piezoelectric materials in tactile sensors due to their light weight, low power consumption, simplicity of fabrication, and flexibility, properties suitable for overlapping large areas and curved surfaces. Moreover, PVDF films that can measure pressure directly are able to generate a dynamic response with high sensitivity in dynamic environments with which robotic tactile applications are always confronted and in which the bandwidth of the response frequency is substantially high, about 0–1 kHz [133]. 

In human skin, many tactile corpuscles, such as Pacinian corpuscles, Ruffini corpuscles, and Meissner’s corpuscles, are capable of detecting stimuli of different frequencies (from 0 to 700 Hz). These tactile corpuscles can be simulated by piezoelectric tactile sensors for their individual dynamic sensing characters.

**Figure 16:**
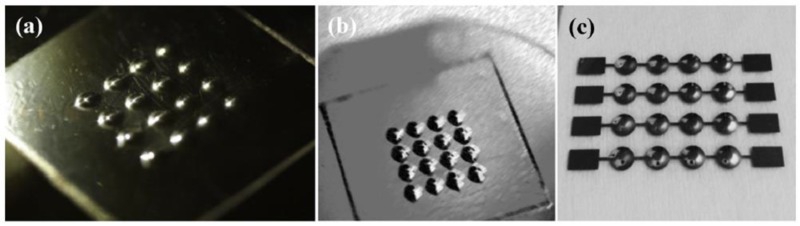
(**a**–**c**)The optical photographs of the fabricated dome-shaped polyvinylidene fluoride (PVDF) film and tactile sensors. Reprinted from [134], copyright (2014), with permission from Elsevier.

**Figure 17:**
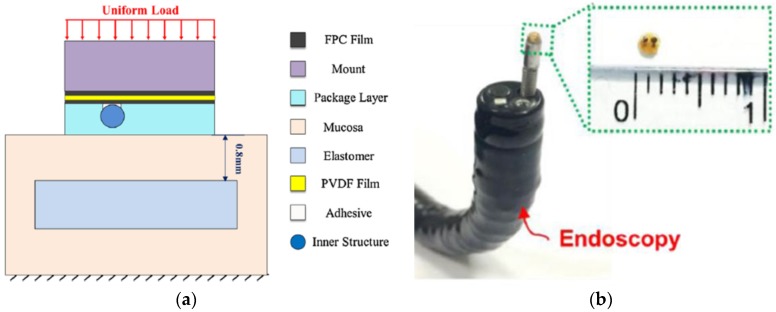
(**a**) Structure view of the proposed tactile sensor. (**b**) Image of the miniaturized tactile sensor (Ø = 1.5 mm) mounted on an endoscope. Reprinted from [135] copyright (2016), with permission from Elsevier.

In recent years, many researchers have proposed piezoelectric tactile sensors consisting of piezoelectric materials such as PVDF to mimic dynamic tactile mechanoreceptors in human fingertips. Seminara et al. developed arrays of piezoelectric polymer transducers that can be used in the large-area implementation of flexible artificial skin in an operation frequency range of 1 Hz–1 kHz [68]. The proposed technology and feasible substrate enable one to mold a tactile array into any shape with good reproducibility and low cost. Kim et al. proposed dome-shaped piezoelectric tactile sensor arrays utilizing a controlled inflation technology, and the shape of the cells in the PVDF film could be easily changed during the fabrication process [134]. To minimize the influence of crosstalk affecting piezoelectric tactile sensors composed of discrete sensing elements in an array form, they utilized dome shape cells as shown in Figure 16; thus, the tactile sensors had a higher sensitivity than conventional flat tactile sensors. The screen-printing technique was adopted due to the PVDF film’s poor performance. Chang et al. developed a piezoelectric tactile sensor using a PVDF sensing film and two components made of PDMS to detect submucosal tumor in endoscopy [135]. Such tactile sensors can provide effective detection of submucosal tumors and lesions due to their individual structures (as shown in Figure 17) in comparison with traditional endoscopy sensors, which cannot detect gastrointestinal mucosal tumors effectively in early stages. Spanu et al. presented a piezoelectric tactile sensor based on PVDF and an organic transistor (organic charge modulated field-effect transistor (OCMFET)) with a high sensitivity [133], and Maita et al. developed a flexible piezoelectric tactile sensor composed of a poly[vinylidenefluoride-co-trifluoroethylene] (PVDF-TrFE) capacitor according to an extended gate configuration [69]. These two sensors are flexible, sensitive, and suitable for large-area applications, which is important for the fabrication of industry tactile sensors. Moreover, in comparison with conventional organic FETs, these sensors can detect fast varying contact forces due to their much higher mobility. 

In addition to detecting external forces, procuring information about surface texture is also an important part in both human and robotic tactile sensing, as it is critical for distinguishing contact objects. Roughness is considered the most critical feature of a surface texture [136]. Liu et al. proposed a piezoelectric tactile sensor array with PVDF for roughness detection utilizing the interval of response time between adjacent sensor units and the principal frequency of vibration at different scanning velocities [71]. By measuring principal frequency and scanning velocity, the surface spatial period can be calculated; meanwhile, another characteristic variable texture amplitude can be extracted according to the amplitude of the output charge in the measurable range. Thus, the roughness of a stimuli’s surface texture can be completely determined. 

However, piezoelectric tactile sensors also have inherent drawbacks, the greatest one of which is the inability to measure static contact forces because the induced charge in piezoelectric materials dissipates very quickly. This means piezoelectric tactile sensors cannot be effective in static environments. Additionally, robustness and sensitivity to temperature are lacking. 

### 2.5. Optical Tactile Sensors

Optical tactile sensors obtain tactile information by analyzing changes of internal or output light. In optical tactile sensors, optical fibers are utilized as a medium for transmitting light, so the advantages of optical fibers can be regarded as merits of optical tactile sensors to some degree. Advantages of optical-based tactile sensors include a light weight, physical flexibility, chemical inertness, a fast response, and a small size. In addition, optical tactile sensors are immune to electromagnetic interference, which means they are compatible with magnetic resonance imaging (MRI) and can be integrated with minimally invasive surgical (MIS) manipulation tools [72]. As for optical tactile sensors in the form of arrays, there is no interconnection between optical fibers and no parasitic disturbance due to the property of passive electricity, which is another advantage over capacitive-based and piezoelectric tactile sensors.

The sensing principles that optical tactile sensors depend on include light intensity modulation, fiber Bragg grating (FBG) technology, and interferometry detection, the first two of which are mostly utilized.

#### 2.5.1. Light Intensity Modulation

This principle is mostly exploited in optical tactile sensors. Tactile sensors based on light intensity detection detect tactile information, such as contact forces and the position of forces, by measuring the change in light intensity or the optical power at the output side of the optical fibers, which are generally results of fibers bending when contact forces are applied to them. 

**Figure 18:**
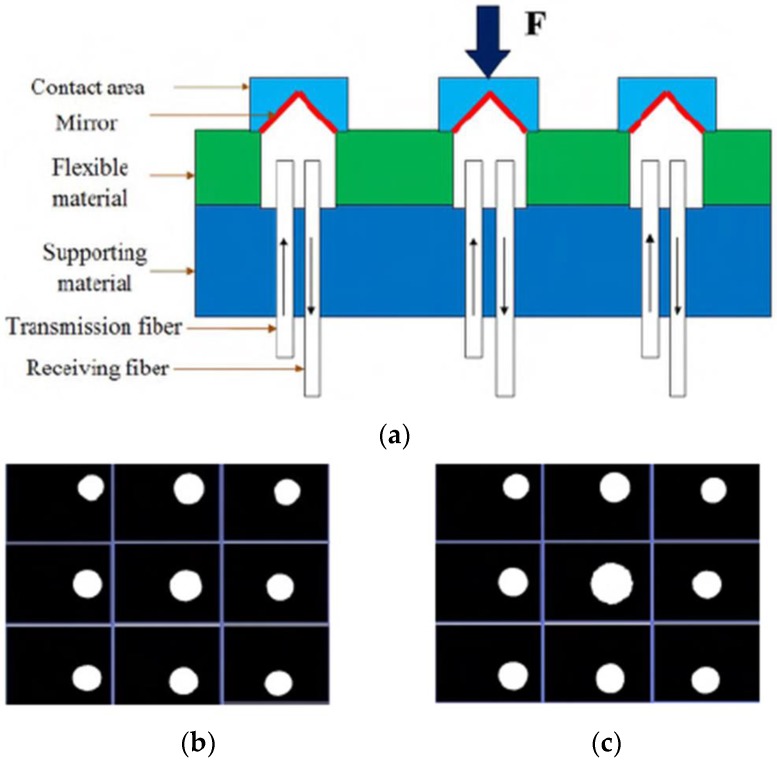
(**a**) Design of tactile sensor with mirror placement. (**b**) Video output with unload status. (**c**) Video output with force applied in central element © [2012] IEEE. Reprinted, with permission, from [72].

Ahmadi et al. proposed a beam-type optical tactile sensor that can be used in minimally invasive robotic surgery [73]. The tactile sensor they developed is, by measuring the power loss in each fiber, capable of measuring both the value and the probable position of contact forces. Meanwhile, the optical power and the size of the fabricated sensor are still pretty high. Xie et al. proposed an optical tactile sensor array that can only measure normal contact forces with mirrors [72]. As shown in Figure 18, when one sensing element is pressed by a normal force, the light intensity in the receiving fiber increases, and the corresponding pixel is hence activated in the output camera video.

#### 2.5.2. Fiber Bragg Grating (FBG) Technology

**Figure 19:**
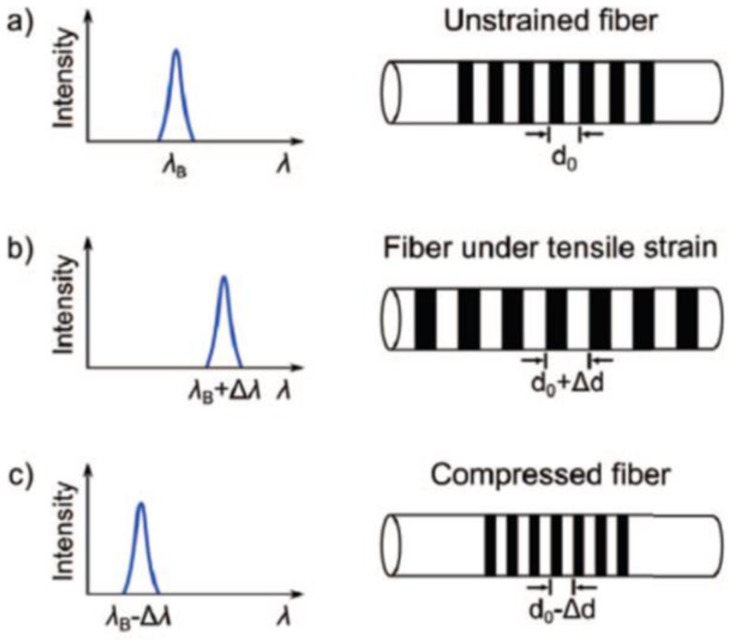
Spectra of FBGs reflecting light under strain or compression. (**a**) Without strain or compression; (**b**) the reflected wavelength is shifted to higher wavelengths under strain; (**c**) the reflected wavelength is shifted to lower wavelengths under compression © [2012] IEEE. Reprinted, with permission, from [138].

The fiber Bragg gratings inscribed in the cores of optical fibers work like a dielectric mirror as a narrow bandwidth of light in the fibers. Most of the transmitting light is diffracted and returns to an inverse direction, with only certain wavelengths passed through. The wavelength range of light reflected by fiber Bragg gratings in fibers is determined by the effective refractive index of the fiber core and the spatial period of the grating. Forces applied to the FBG tactile sensor can change both the intervals of gratings and the refractive index, which will result in a Bragg wavelength shift of output light. By measuring the signal of the wavelength shift instead of the light intensity, the tactile information can be extracted. Thus, FBG tactile sensors do not have a problem of intensity fluctuation and are able to constitute an array with two fibers, at least as several gratings can be inserted in the same fiber [137]. Ledermann et al. proposed optical tactile sensors using FBG technology to sense tactile perceptions and shapes for MIS [138]. Their tactile sensor is able to measure external strain in a high solution by analyzing the reflected light spectrum, as shown in Figure 19. One fiber Bragg grating tactile sensor array introduced by Song et al. in [139] is implanted in flexible silicon. This array has a good dynamic response and is suitable for large-area fabrication because of its high flexibility, but the size needs further miniaturization. 

The sensing range of FBG tactile sensors is very broad, and such sensors have much potential in multidimensional robotic tactile applications and MIS applications [140]. However, tactile sensors based on FBG technology and other optical principles are susceptible to temperature influence, which means the compensation must be taken into consideration in the design. Moreover, light loss occurs due to the micro-bending of fibers, and methods of computation in analysis tactile information are relatively complex, which are disadvantages of optical tactile sensors.

## 3. Applications

During the past two decades, tactile sensors have been applied to many fields, such as robotics and biomedicine. Here, we focus on robotic applications, discussing tactile sensors used in robots and MIS (minimally invasive surgery), respectively.

### 3.1. Robots

The robot’s independent exploration is achieved by actions of various manipulators, which are called exploration procedures (EPs). Lederman [141] defines eight kinds of EP actions for the robot’s active exploration, which regulate the robot exploration action and are the foundation for achieving rapid identification. The purpose of tactile exploration is to obtain environmental information and information about the objects to be manipulated in unstructured environments, such as the hardness, shape and position of objects. Furthermore, object property information can also be acquired by active exploration based on tactile sensing. For example, Chitta et al. [142] utilized the robot to capture objects in order to obtain the relationship between applied forces and the deformation. Meanwhile, they designed a rotation-based EP strategy to identify the internal information of objects (e.g., whether it is hollow or not, whether there is liquid or not, and so on). 

With respect to tactile recognition algorithms, Spiers et al. [143] achieved object identification and feature extraction by analyzing robot end position data and tactile sensor data based on the machine learning technique (random forests) and parametric methods. This approach is tolerant of the relatively low accuracy of tactile sensors and robots, so it has better adaptability. Liu [144] proposed a tactile recognition method based on sparse coding of joint kernel, which solved the problem of tactile information interference between multiple fingers of dexterous hands when they contact objects at the same time. Luo [145] used the tactile sensing array to obtain touch images when objects are touched, and proposed feature description based on the SIFT (scale-invariant feature transform, an algorithm in computer vision to detect and describe local features in images) descriptor to realize the shape recognition of objects. Mohsen et al. developed an integrated tactile sensor that consists of an accelerometer, a proximity sensor, a temperature sensor, and a force sensor [146], and they proposed a recognition framework for unknown objects based on a variety of tactile perceptions of information [147]. 

It is obviously far from sufficient to obtain features of objects relying solely on tactile sensors in unstructured environments, so many researchers have combined touch sensors with other sensors for improved exploration and recognition results. Sun et al. combined tactile sensors with deep vision to make the recognition result more accurate [148].

In the case of tactile-based robot control, Song et al. [149] proposed a two-finger dexterous hand stabilization control method based on tactile force feedback, which determines the optimized grasp angle and minimizes the possibility of falling in the gripping process to enhance the stability of object grasp. Additionally, Benjamin et al. [150] achieved the classification, identification, and crawling for cylinders of different diameters utilizing the GR2 hand claw platform with tactile feedback and a Bayesian identification method. 

### 3.2. MIS

**Figure 20:**
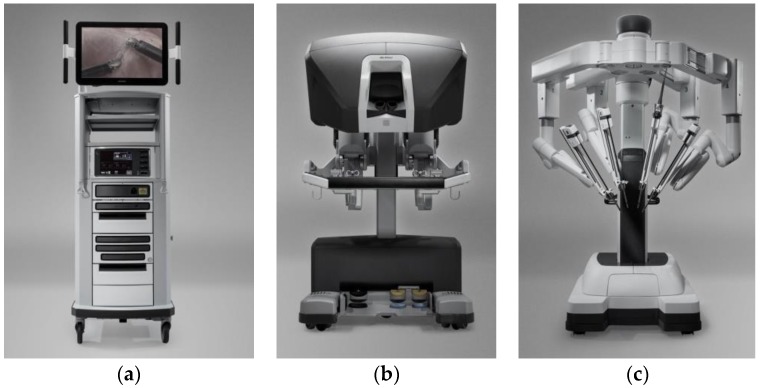
Three major components of the da Vinci Surgical System: (**a**) vision system; (**b**) surgeon console; (**c**) patient-side cart. Reprinted from [153].

The application of tactile sensors in MIS gained prominence in the 1990s [25], and rapid developments have occurred in the past two decades. In comparison with conventional manual operation, much smaller incisions result in significantly reduced intraoperative blood loss, tissue trauma, and risk of post-operative infection [140,151]. Thus, patients can undergo less pain and a faster recovery time. For instance, in laparoscopy, the surgeon inserts a miniature camera and a set of elongated slim tip-mounted tools through narrow puncture openings (between 3 and 12 mm) in the abdominal wall of the patient [12,152]. Minimally invasive robotic surgery systems have attracted significant interest from both industry and academia. Among the commercial available products, the da Vinci Surgical System is typical. Figure 20 shows the three major components of the da Vinci Surgical System: the vision system, the surgeon console, and the patient-side cart [153].

**Figure 21:**
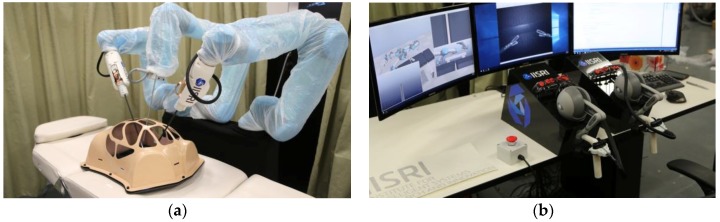
(**a**) The manipulation of HeroSurg; (**b**) the workstation console of HeroSurg. Reprinted from [161].

However, the lack of tactile information limits the performance of the surgical system. Tactile sensing can offer the surgeon the ability to measure the magnitude of force applied to the tissue, and dissect the characteristics of the tissue, which is important for palpation during surgery. To solve this problem, researchers have developed various tactile sensors with different mechanisms mounted on the tip of slim tools. One of the main kinds of instruments is of a probe shape, which is suitable for optical tactile sensors [154,155,156]. Optical fibers can be easily placed into metal conduits to reduce wiring complexity. Another main kind of instrument is the clamp, which is used to clamp the tissue for suturing or diagnosis. Piezoresistivitive, capacitive, and piezoelectric tactile sensor arrays can be mounted on the flat part [157,158,159,160]. Nonetheless, the surgical system with tactile sensing ability is still unusual. Engineers from Deakin University and Harvard University have developed a new type of robotic surgical system with a sense of touch in 2016, namely the HeroSurg [161]. The manipulation and workstation console are shown in Figure 21.

## 4. Future Directions

Up to now, single tactile sensors with diverse transduction mechanisms have been adequately researched and a large number of promising sensors have been reported in the literature [162]. Compared to the research on tactile sensing systems, there are relatively more studies on single tactile sensors with new materials and novel structures. The performance of some proposed sensors even extend beyond human sensing capabilities. However, customized sensing arrays and systems are still urgently required, mainly because there has been little attention on robustness, which has been the greatest impediment to practical application scenarios. Most of the reported tactile sensors are very sensitive to the working environment, such as temperature, humidity, and unexpected collisions. Consequently, insulated packages of the flexible sensing arrays and materials insensitive to temperature and humidity should arouse researchers’ attention.

The consistency is another critical issue in tactile sensing arrays. As the number of sensing elements increases, it becomes extremely time-consuming to calibrate every single element each time the array is used. Hence, there should be good consistency between every two elements, including the zero-load output and the pressure response curve. Moreover, the tactile sensing array should maintain good consistency in at least thousands of iterations. At present, most of the tactile sensors cannot meet these criteria.

The application scenarios of tactile sensing arrays could be further broadened, especially in medical treatment and robotics. As artificial intelligence (AI) has made tremendous progress in recent years, robotic applications have also undergone great strides. Robots are becoming increasingly anthropomorphic and intelligent. There are more research foci on the combination of basic sensory systems, in which the fusion of tactile and visual sensations are a very promising research direction. For instance, robotic grasps, which is a basic, but significant research focus, require tactile and visual information jointly. At present, most studies concentrate on detecting the object and grasp location with visual information and then use tactile feedback to determine if the object has been caught. We could further integrate visual and tactile information, not limited to catching an object. Tactile sensors could be used to feel the veins, texture, and hardness of objects. Coordinating with the shape and color information obtained by visual sensors, robots could have a more comprehensive understanding of the objects it contacts, just as infants do.

The application of tactile sensors in the medical field is mainly focused on the clips of surgical robots that judge the degree of hardening and lesions of the contacted tissues and are stuck to the human body to detect physiological signals. This is far from satisfying the demands of doctors. Tactile sensors need to be smaller and more sensitive to meet the needs of more sophisticated surgery. For instance, cardiologists need tactile sensors to detect blood vessel contraction tension when bypass surgery is performed. Remote surgery is a highly meaningful research that will allow people all over the world to share quality physician resources. Tactile sensors could be used to feed the tactile information detected by the surgical robot to the far-end doctor. The robot could receive certain stimuli through the actuator and detect diseased tissue more intuitively. The same closed-loop control strategy could be used to study prostheses to help people with amputations.

## 5. Conclusions

A general introduction to human tactile systems and the development of tactile sensors since the 1970s has been provided in this paper. We highlight state-of-the-art design trends with diverse transduction mechanisms, including capacitive, piezoresistive, piezoelectric, inductive, and optical sensors. Moreover, the advantages and disadvantages of the mentioned mechanisms are analyzed. Capacitive and piezoresistive tactile sensors are widely used in robotic triaxial tactile sensing due to full-blown techniques and excellent performance. To achieve the flexibility of human skin, CNT-based nanocomposites are an excellent choice. Their low cost and easy fabrication processes, such as screen printing, are also suitable for large-scale industrial production. Due to obvious drawbacks, piezoelectric, inductive, and optical tactile sensors are not predominant choices for robotic applications.

Although there are some commercially available products, such as force sensing resistors (FSR) [107,108] and finger-shaped BioTac sensors [163], customizable and modular tactile sensing arrays, suitable for diverse surfaces, are urgently needed. The market still lacks system-level products. To close the gap between academia and industry, researchers should pay more attention to the robustness and consistency of the tactile sensors. Moreover, the application scenarios of tactile sensing arrays in medical treatment and robotics could be further broadened. The fusion of visual and tactile information and using tactile feedback to form closed-loop controls are promising and meaningful research directions.

## Figures and Tables

**Table 1:** State-of-art tactile sensors.

Year	Author	Sensing Principle	Miniaturization Technique	Force/Pressure Sensitivity ^1^	Range of Force+(N) ^2^/Pressure * (kPa)	No. of Sensing Element
2012	Chien-Chun Chen et al. [58]	Capacitive	—	14%/kPa	2+/20 *	4 × 4
2013	J. A. Dobrzynska et al. [59]	Capacitive	MEMS on Polymer	2.4%/kPa (nf, 0–10 kPa) 0.066%/kPa (nf, 10–140 kPa) 0.028%/kPa (shf)	140 *	2 × 2
2014	Benjamin C.K. Tee et al. [60]	Capacitive	MEMS on Polymer	—	10 *	13 × 10
2015	Alexi Charalambides et al. [61]	Capacitive	MEMS on Si	190 mN (nf) 50 mN (shf)	8 + (nf) 2 + (shf)	2 × 2
2015	Guanhao Liang et al. [62]	Capacitive	MEMS on Polymer	58.3%/N(x) 57.4%/N(y) 67.2%/N(z, 0–0.5 N) 7.7%/N(z, 0.5–4 N)	0.5 + (x, y) 4 + (z)	4 × 4
2016	Axaykumar Rana et al. [63]	Capacitive	MEMS on Si	—	15+	3 × 4
2012	Kentaro Noda et al. [64]	Piezoresistive	MEMS on Si	0.17%/kPa	−1.8–1.8 *	1
2013	Xinchuan Liu et al. [65]	Piezoresistive	MEMS on Polymer	23%/kPa	6.67 *	1
2013	Rohit Kilaru et al. [66]	Piezoresistive	MEMS on Polymer	8.05%/N(nf)	—	1
2014	Soonjae Pyo et al. [67]	Piezoresistive	MEMS on Polymer	6.67%/N(nf) 86.7%/N(shf)	2+/163 *	2 × 2
2013	Lucia Seminara et al. [68]	Piezoelectric	MEMS on Polymer	—	8+	12
2015	Francesco Maita et al. [69]	Piezoelectric	MEMS on Polymer	430 mV/N	2+	1
2017	Minkyung Sim et al. [70]	Piezoelectric	Nanotechnology	—	275 *	3 × 3
2017	Weiting Liu et al. [71]	Piezoelectric	MEMS on Si	—	2+	2 × 2
2012	Hui Xie et al. [72]	Optical	—	—	—	3 × 3
2012	Roozbeh Ahmadi et al. [73]	Optical	MEMS	—	4+	1
2013	Alessandro Massaro et al. [74]	Optical	MEMS on Polymer	—	3.9+	1
2017	Eric Fujiwara et al. [75]	Optical	—	0.08 N	0.5+	1
2012	S. Wattanasarn et al. [76]	Magnetic	MEMS on Polymer	0.68 mV/N	2.5+	1
2015	Ahmed Alfadhel et al. [41]	Magnetic	Nanotechnology	856 mΩ/kPa	0.85 *	1

^1^ nf: normal force; shf: shear force. ^2^ +: range of force (N); *: range of pressure (kPa).

**Table 2:** Transduction mechanisms and their relative advantages and disadvantages [77,78,79,80].

Transduction Mechanisms	Advantages	Disadvantages
Capacitive	High sensitivity High spatial resolution Large dynamic range Temperature independent	Stray capacitance Complex measurement circuit Cross-talk between elements Susceptible to noise Hysteresis
Piezoresistive	Simple construction High spatial resolution Low cost Compatible with VLSI	Hysteresis High power consumption Lack of reproducibility
Piezoelectric	High frequency response High accuracy High sensitivity High dynamic range	Poor spatial resolution Charge leakages Dynamic sensing only
Optical	Good reliability Wide sensing range High repeatability High spatial resolution	Non-conformable Bulky in size Susceptible to temperature or misalignment
Inductive	Linear output High sensitivity High power output High dynamic range	Low frequency response Poor reliability More power consumption
